# Editorial: Early chest drain removal following lung resection

**DOI:** 10.3389/fsurg.2023.1185334

**Published:** 2023-03-31

**Authors:** Marco Scarci, Andreas Gkikas, Davide Patrini, Fabrizio Minervini, Robert J. Cerfolio

**Affiliations:** ^1^ Department of Thoracic Surgery, Imperial College Healthcare NHS Trust, London, United Kingdom; ^2^ Department of Thoracic Surgery, University College London Hospitals, London, United Kingdom; ^3^ Department of Thoracic Surgery, Cantonal Hospital Lucerne, Lucerne, Switzerland; ^4^ Department of Cardiothoracic Surgery, New York University Langone Health, New York, NY, United States

**Keywords:** chest drain, chest drain management, chest drain protocol, lung canc er (LC), thoracic

## Introduction

Thoracic surgeons have pioneered fast-track and Enhanced Recovery After Surgery (ERAS) guidelines over 22 years ago (1) and have developed new peri-operative techniques and pathways that ensure high quality post-operative results and quicker patient recovery. This is reflected on the constant pursue of creating new surgical approaches, establishing the Enhanced Recovery After Surgery (ERAS) guidelines and the unceasing re-evaluation of pre-operative assessment and post-operative management (2–4).

For lung resection surgery in particular, minimally invasive thoracoscopic methods have gained ground since 1990. The most prominent approaches in this category are the video-assisted and completely portal robotic techniques, VATS and CPR respectively (5). According to a recent randomized controlled study, evidence supports that VATS is associated with less post-operative pain, complications and length of hospital stay compared to an open approach (3).

Another aspect of thoracic surgical practice that has been a topic of debate is chest drain management. This is considered one of the main causes of long hospital stay, post-operative pain and complications. Overall, prolonged use of chest drain increases pain, reduces patient mobility and causes complications. In this review, we summarize the existing publications in early chest drain removal and no chest drain insertion practice following lung resection *via* VATS. We subsequently commented on the effectiveness and safety of early chest drain removal strategies following open and robotic-assisted approaches.

## Methods

We performed a review of the literature and searched the medical electronic libraries of Medline and Cochrane using the search strategy presented in Appendix 1.

Our inclusion criteria were studies that investigated early chest drain removal strategies or no chest drain insertion strategies post VATS lung resection. We excluded studies that investigated only open approaches, focused solely on pediatric population, and were published in any language other than English.

Our search strategy returned 2,115 studies which were screened. After title and abstract and full text screening, we found 41 studies that met our criteria (6–46). We classified the eligible studies in three distinct categories: (a) studies that investigated early chest drain removal in non-anatomical lung resections, (b) studies that investigated no chest drain insertion post lung resection and (c) studies that investigated early chest drain removal in anatomical lung resections. We defined the no chest drain insertion group as the absence of chest drain before patient extubation. The outcomes that we were interested in were the length of hospital stay, the post-operative complications and more particularly the events of pneumothorax and pleural effusions post-operatively, the incidence of chest drain insertions and the post-operative pain assessment. We present the results of these studies in the form of narrative text.

## Results

### Early chest drain removal in VATS non-anatomical lung resections

We found four studies that met our criteria for early chest drain removal after non-anatomical lung resection (6–9). Three of these studies were published between 1998 and 2006 and are all case series following VATS wedge resections (6–8). The enrolled patients had peripherally located disease and were all performed under single-lung ventilation. The investigated strategies did not focus on intra-operative air seal tests but re-inflation of the lung was reviewed under direct vision before closure. The size of chest drains was between 24–28 Fr and in two studies, suction was applied at the end of the operation (−15 cm H_2_O to −20 cm H_2_O) (7, 8). The definition of early chest drain removal varied among the three publications from 60 min post-operatively in the study by Fibla et al. to 8 h post-operatively by Chang et al. (6–8). However, all of them had as initial goal for chest drain removal while the patient was in Recovery. The decision for early removal was determined on the absence of air leak, drainage of <50–100 ml/hr and good lung expansion on post-operative CXR.

In the group of patients who underwent early chest drain removal their length of stay (LOS) was shorter compared to those who had to either keep their chest drain because they did not meet the removal criteria (7) or because they were managed with more conservative chest drain strategies (6). The incidence of new chest drain insertion among patients who had their drain removed early was 2% (3/146) (8), 2% (1/45) (7) and 0% (5). Only one study investigated the effect of their strategy on post-operative pain and showed that the total narcotic requirement was significantly less in the early removal group (*p* = 0.005) (5).

The fourth study after non-anatomical lung resection was solely focused on patients who underwent double-lumen tube intubation and VATS bullectomy, mechanical and chemical pleurodesis for primary spontaneous pneumothorax (9). This included 105 patients who had a negative air leak test intra-operatively. The chest drain was removed on post-operative day 0 in all patients after absence of air leak during cough, absence of bloody discharge and completely expanded lung on immediately post-operative CXR. Their mean length of stay was 1.1 ± 0.5 days, one patient required chest drain re-insertion because of pneumothorax. 12 patients (11.4%) experienced recurrence of their pneumothorax after having had their operation. The investigators also reported that among 18 patients from their institution who had the same procedure in that period but did not have their chest drains removed on day of the operation, due to air leak and bloody discharge, the recurrence of pneumothorax was estimated at 11.1% (2/18), *p* = 1.00.

### No chest drain insertion in VATS non-anatomical lung resections

We identified 26 studies that investigated management strategies that used no chest tube after VATS non-anatomical lung resections (10–35). From these studies, 85% (22/26) have been published after 2016 which illustrates the growing interest of thoracic surgeons on the topic of no chest drain following thoracic procedures (14–35). These are 7 randomized controlled trials, 15 retrospective studies and 4 prospective studies.

Following review of their inclusion and exclusion criteria, we concluded that the patients who were enrolled for the no chest drain management strategy were a highly selected population. The selection criteria were understandably variable among the different studies but they mostly comprised of patients with no diffuse underlying lung disease, who were not coagulopathic, had no intra-operative complications, no extensive adhesions, no unstable systematic diseases such as active infection, uncontrolled diabetes, hypertension or angina, no previous ipsilateral thoracic surgery, no recent (<3 months) radiotherapy or chemotherapy and did not undergo anatomical lung resection or conversion from VATS to an open approach.

Furthermore, in all these studies the investigators performed an intra-operative air leak assessment after lung resection. Some studies assessed the presence of air leak by immersing the lung in saline and then proceeding with re-inflation of the operated lung with positive inspiratory pressures (ranged from −10 cm H_2_O to 30 cm H_2_O) and assessing under direct vision for sites of air leak (17, 18, 25, 26).

### Chest tube removal prior to leaving the operating room

Another method which was widely performed was the temporary insertion of a chest drain inside the pleural cavity (10, 11, 22, 24–28, 30–33). After closing all the intra-operative chest wall incisions, the drain was subsequently either connected to a digital suction system with negative suction (24, 27, 28, 30, 31, 35) or its extra-thoracic end was simply immersed to a bowl with sterile water (10, 11, 22, 32) while the anesthetic team administered manually positive inspiratory pressures. Then if there was no air leak by both the anesthesiologist assessment of volume delivered and returned and by the digital air leak meter attached to the chest tube the chest drain was removed before extubation. In some studies, the investigators decided to leave either a central line or an ABLE catheter within the chest as a safety net to evacuate excess air of fluid if required (21, 22, 32, 33). The size of the temporary chest drain ranged from 12 Fr to 24 Fr while in two cases the researchers used a nasogastric tube (19) and a Ryles' tube (11) to assess intra-operatively the presence of air leak.

When this highly selected criteria were met and the patients did not have a chest drain inserted in the operating theatre, their length of stay was significantly shorter in 16/17 studies compared to patients who had chest drain inserted (10–14, 17–22, 25–27, 30, 33). This was confirmed even from the randomized trials in which the enrolled patients met the same intra-operative criteria during the air leak test (11, 17, 20, 22, 26, 27). The incidence of pneumothorax during the post-operative period was higher among the non-chest drain group but was statistically significant in 4 studies (18, 20, 26, 32). However, this significance was not translated in chest drain insertion rate between the two groups at any study (10–23, 25–27, 32–34). Furthermore, the no chest drain strategy appeared to improve significantly the post-operative pain in 10 studies (14, 18, 20, 22, 23, 25–27, 32, 33) which was more significant on post-operative day 0 and day 1, 2 and 3. The only study that assessed post-operative pain for longer period (in 1 week and 1 month) did not show significant differences between chest drain insertion and no chest drain insertion groups (25).

### Early chest drain removal in VATS anatomical lung resections

We identified 11 studies that investigated early chest drain removal strategies following VATS anatomical lung resection (36–46). All of them were published mostly over the last decade, with the oldest study on the topic being from the United States in 2007 (36). These are comprised of 2 randomized controlled trials, 1 prospective multi-institutional cohort study, 1 prospective single-institutional cohort study and 7 retrospective studies and case series.

The population enrolled in these studies excluded patients who had chemotherapy and radiotherapy, patients who were converted from VATS to open, patients with history of previous thoracotomy, heart failure, nephritic syndrome, chronic renal failure, cirrhosis and patients with extensive pleural adhesions. In 4 studies, an intra-operative air leak test was performed (38, 42, 45, 46). If areas of air leak were identified during the test then pneumostasis was attempted by using polyglycolic acid (PGA) mesh, fibrin glue (38, 42), staplers (45) or continuous suturing of the pleural edge of the preserved segments (45, 46). The only study that investigated the effect of pneumostatic agents in early drain removal, applied PGA mesh and fibrin glue on the areas of intra-operatively identified air leak (38). They compared 133 patients who received these agents with 73 patients who did not. This showed that the duration of chest drain and recurrence of air leaks was no different between these two groups. However, it is important to note that the patients who did not receive pneumostasis did not have identifiable air leak during the operation.

From the 11 studies, 1 had a protocol of connecting the chest drain on suction after the operation (38). The earlier chest drain removal following VATS lobectomy or segmentectomy was attempted by Murakami et al. who aimed to take the drain out just after extubation (42). Following PGA mesh and fibrin glue application on the identifiable areas of air leak during a water seal test, the decision was made for early chest drain removal strategy if no further air leak was seen. At the end of the procedure, a 20 Fr chest drain was temporarily inserted and connected to −5 cm H_2_O suction while simultaneously, the lungs were inflated by continuous positive pressure at 10 cm H_2_O by the anesthetist. The drain was successfully removed in 102 (63%) of the 162 patients. No patient required chest drain insertion for pneumothorax or surgical emphysema. One patient out of 102 underwent a needle puncture for the drainage of persistent pleural effusion on post-operative day 10. There was statistically significant reduction in VAS values on pain assessment from post-operative day 0 (*p* < 0.001) until day 3 (*p* < 0.05) when compared to the patients who kept their chest drain *in situ* after extubation.

Pfeuty and Zheng aimed at removing the chest drain in <24 h after the operation (45, 46). Their criteria were <20 ml/min air leak for 4 h, with no hemorrhagic or chylous drainage despite the output. Another study in which the investigators decided on drain removal regardless of the amount of pleural drainage was the one by Ueda et al. (38). Although, they did not specify a specific post-operative day for early drain removal. Xing defined as early chest drain removal the first 48 h from the operation (44). Their criteria were no atelectasis on post-operative day 1 CXR, absence of air leak and no purulent or chylous discharge. Similar thresholds were used by 2 more studies (41, 43) who aimed at removing the chest drain on post-operative day 1. However, in one of these studies the investigators were keeping a smaller (7 Fr) drain *in situ* until day 3–4. This drain had been inserted intra-operatively. In four publications, the authors used variable thresholds on tube drainage (36, 37, 39, 40). The cut off for drain removal in these studies ranged from 300 to 500 mls/24 h.

Even in studies that did not use the daily drain output as a criterion for drain removal, there were not many reported cases that required chest drain re-insertion (2.2%, 18/802) (40, 42, 44–46). There were three studies that evaluated the effect of early drain removal on post-operative pain (42, 45, 46). In two studies (42, 45), early removal was associated with less pain on VAS while the third study did not reflect the same outcome (46). The latter study assessed the VAS of pain on day 0, 1, 7 and 1 month after the operation and showed no significant differences in the postoperative pain between >24 h and ≤24 h drain removal (46).

## Comment

### Early chest drain removal in open lung resection

The benefits from adapting early chest drain removal protocols even in lung resections performed *via* thoracotomy was highlighted by Nomori et al. from 2001 (47). Their findings supported that in absence of air leak and drain output of less than 400 mls, the chest drain can be safely removed on post-operative day 1. This was achieved in 60% of their patients (25/42). When they compared their new protocol with a historic control group from their database, the percentage change in 6-minute walk test 1 week post-operatively was significantly higher in the early chest drain removal group.

The same threshold of 400 mls over 24 h and absence of air leak was used by Bertholet et al. for early drain removal after lung resection for lung cancer *via* posterolateral thoracotomy (48). In this study, the early removal group was also compared to a historic cohort from the same department when two chest drains were inserted instead of one. The criteria for drain removal in the old protocol were absence of air leak and drain output of less than 150 mls over a day. The patients from the early removal group had a significantly reduced hospital stay (11 from eight days) with no statistical differences being observed in terms of postoperative complications. Three patients (3/68, 4.4%) from the historic cohort and six (6/65, 9.2%) from the new protocol required pleural puncture due to pleural effusion post drain removal (*p* = 0.318).

### Early chest drain removal in robotic-assisted lung resection

A recent meta-analysis has demonstrated that robotic-assisted lung resection for lung cancer is a safe alternative to VATS with lower conversion rate and shorter length of hospital stay (49).

Geraci and Cerfolio from NYU Langone recently reported a prospective protocol on the removal of all chest tubes within 6–8 h after robotic pulmonary lobectomy and segmentectomy on 253 consecutive patients. The goal was for all patients to go home in 24 h (with a chest tube attached to a digital air leak device) and or to have their tubes removed within 6–8 h after resection. Patients were given ice-cream in the recovery room to rule out a chylothorax to facilitate early chest tube removal. The authors showed that it is feasible and safe to discharge patients on post-operative day 1 (50). Overall, this was achieved in 53% (134/253) of the patients and it reached 97% (28/29) and 68% (23/34) during the last quartile of the study for segmentectomy and lobectomy respectively. The criteria for chest drain removal were solely based on absence of air leak on a digital air leak meter with a fully expanded lung or fixed pleural space deficit on post-operative CXR regardless of fluid output. From this approach and efficient operation with little to no blood loss and under two hours of total operative time tehre were no conversions to thoracotomy, only 2.4% (6/253) of the patients had major complications with 3 of them requiring outpatient thoracentesis because of pleural effusion within 30-days. There was no 30- or 90-day mortality while only 1.6% (4/253) required re-admission within 30-days.

## Conclusion

Evidence supports that early chest drain removal strategies can be beneficial to certain patients following lung resections. Video-assisted and robotic-assisted approaches offer the most favorable outcomes when such protocols are adopted. These appear to reduce length of hospitalization and are associated with less pain during the first post-operative days. Even the more ambitious practices of no intra-operative chest drain insertion has shown promising results and appear to be safe in highly selected patient population. The ERAS guidelines in thoracic surgery have not yet addressed the significance of intra-operative assessment of patients who may benefit from such strategies (4). We believe that further research is required in order to identify accurately the subgroup of patients who benefit from these practices.
